# No Success without Effort: Follow-Up at Six Years after Implementing a Benchmarking and Feedback Concept for Postoperative Pain after Total Hip Arthroplasty

**DOI:** 10.3390/jcm12144577

**Published:** 2023-07-10

**Authors:** Jan Reinhard, Loreto C. Pulido, Melanie Schindler, Amadeus Schraag, Felix Greimel, Joachim Grifka, Achim Benditz

**Affiliations:** Department of Orthopedic Surgery, University Medical Center Regensburg, Asklepios Klinikum Bad Abbach, Kaiser-Karl-V.-Allee 3, 93077 Bad Abbach, Germany; loreto.pulido@ukr.de (L.C.P.); melanie.schindler@ukr.de (M.S.); amadeus.schraag@ukr.de (A.S.); felix.greimel@ukr.de (F.G.); j.grifka@asklepios.com (J.G.); achim.benditz@ukr.de (A.B.)

**Keywords:** postoperative pain management, benchmarking, total hip arthroplasty (THA), pain management concept, multidisciplinary, QUIPS

## Abstract

Background: Total hip arthroplasty (THA) is still ranked among the operations with the highest postoperative pain scores. Uncontrolled postsurgical pain leads to prolongated hospital stays, causes more frequent adverse reactions and can induce chronical pain syndromes. In 2014, we implemented a standardized, multidisciplinary pain management concept with continuous benchmarking at our tertiary referral center by using the “Quality Improvement in Postoperative Pain Management” (QUIPS) program with excellent results over a period of two years. The initial study ended in 2016 and we aimed to evaluate if it was possible to obtain the excellent short-term results over a period of six years without any extra effort within the daily clinical routine. Materials and Methods: In a retrospective study design, we compared postoperative pain, side effects and functional outcome after primary THA for 2015 and 2021, using validated questionnaires from the QUIPS project. In contrast to the implementation of the pain management concept in 2014, the weekly meetings of the multidisciplinary health care team and special education for nurses were stopped in 2021. Data assessment was performed by an independent pain nurse who was not involved in pain management. Results: Altogether, 491 patients received primary THA in 2015 and 2021 at our tertiary referral center. Collected data revealed significantly worse maximum and activity-related pain (both *p* < 0.001) in combination with significantly higher opioid consumption in comparison to implementation in 2015. Though the patients reported to be less involved in pain management (*p* < 0.001), the worse pain scores were not reflected by patient satisfaction which remained high. While the participation rate in this benchmarking program dropped, we still fell behind in terms of maximum and activity-related pain in comparison to 24 clinics. Conclusion: Significantly worse pain scores in combination with higher opioid usage and a lower hospital participation rate resemble a reduced awareness in postoperative pain management. The significantly lower patient participation in pain management is in line with the worse pain scores and indirectly highlights the need for special education in pain management. The fact patient satisfaction appeared to remain high and did not differ significantly from 2015, as well as the fact we still achieved an acceptable ranking in comparison to other clinics, highlight the value of the implemented multidisciplinary pain management concept.

## 1. Introduction

Postoperative pain management was neglected over the decades [1]. Severe postoperative pain mainly influences patient satisfaction, leads to higher rates of adverse reactions and can induce chronic pain syndromes [2,3,4]. Total hip arthroplasty (THA) is among operations with the highest postoperative pain scores [5]. The number of primary THA in the U.S. is estimated to increase by 284% to 1,429,000 operations in 2040 [6]. Although primary THA has been voted the most successful operation of the century, more than 10% of patients report postoperative dissatisfaction [7,8,9]. Still, the main reason for postoperative dissatisfaction after THA remains chronic pain [8,10]. A lot of studies aim to detect risk factors for chronic postsurgical pain after total joint arthroplasty [10]. In a multicentric prospective study, risk factors for chronical postsurgical pain were imposed. The authors detected orthopedic surgery, preoperative chronic pain and a high percentage of time, bearing severe pain the first 24 h postoperatively as risk factors [11]. These findings were in line with Gentry et al., who showed that patients with preoperative opioid usage have a higher risk for chronic opioid consumption after THA [12]. Despite the large number of studies performed on pain management, existing postoperative pain management is not sufficient [13]. In the daily clinical routine, postoperative pain management is mainly performed by the treating orthopedic surgeon, who only sees the patient briefly during ward rounds [14]. The nurses, the main medical contact for the patient, have limited involvement in pain management.

Consequently, this highlights the urgent need for a targeted, multidisciplinary multimodal pain management concept to prevent chronic pain syndromes [2]. One approach to benchmarking and ameliorating deficiencies in pain management is the implementation of continuous quality improvement (CQI) strategies [15]. Between 2014 and 2015, we implemented a multidisciplinary pain management concept with continuous benchmarking at our tertiary referral center by using the Quality Improvement in Postoperative Pain Treatment project (QUIPS) [15,16]. The QUIPS project demonstrates a major nationwide German initiative, which records and benchmarks postoperative pain [15]. Featuring more than 200 participating hospitals with data sets from more than 600,000 patients, the QUIPS project represents the worldwide largest database for postoperative pain [17]. The concept incorporates the whole health care team and empowers the role of the nurses who see the patients most frequently and therefore represent the main medical contact person. Consisting of close patient contact, a standardized pharmacological pain scheme (see methods), close interdisciplinary work with special educated pain nurses and wider use of regional anesthesia for better pain control, we achieved remarkable improvements over a period of two years. In comparison to 49 anonymized hospitals, we were ranked best concerning maximum pain and patient satisfaction [16]. By continuous benchmarking, deficiencies such as organizational dysfunction and inadequate education of nurses in pain management were detected and erased [16]. In 2016, the initial study ended. Consequently, the weekly meetings of the multidisciplinary health care team and special education for nurses in terms of pain management were stopped. The project is still running to date with minimum effort in the daily clinical routine. However, the initially established pharmacological pre- and postoperative pain management concept is identical to the implementation study.

### Aim of This Study

The implementation study and thereby the weekly meetings of the multidisciplinary health care team as well as special education for nurses ended in 2016. We aimed to evaluate if it was possible to obtain the early great results involving low pain scores and low opioid consumption over a period of six years within the daily clinical routine.

## 2. Methods

Data assessment took place separately for the years 2015 and 2021. In a retrospective study design, we subsequently compared the outcome parameters of the two time points.

Criteria for inclusion were patients receiving primary THA, aged above 18 years and orientated to time and place. Criteria for exclusion were patient’s refusal to participate, disorientation, sedation, cognitive dysfunction or visitors at the time of data assembly. All patients underwent cementless and collarless primary THA at our tertiary referral center. The operation was performed by using a modified Watson–Jones approach without transection of muscular tissue. Patients were put in the lateral position. Via an anterolateral mini-incision, the intermuscular plane between tensor fascia lata and gluteus medius was used. The advantages lay in the preserved integrity of the surrounding muscles of the hip joint as well as the intact posterior capsule which hinders posterior dislocation [18].

### 2.1. Initially Established Pain Management Concept

From 2014 to 2015, a multidisciplinary pain management concept with continuous benchmarking for patients receiving primary THA was implemented at our university medical center [16]. In line with the implementation study, a multidisciplinary health care team of surgeons, anesthesiologists, nurses and physiotherapists met weekly to detect problems and ameliorate pain management. During these meetings, the health care team was informed about the results and possible improvements were discussed. A lot of deficiencies were of organizational issues. The detected lack of staff was not possible to bear; therefore, the existing nurses received special courses in pain management, involving pharmacological dynamics and non-pharmacological treatment according to the German guidelines of pain management. The courses were held by the treating physicians. The physicians and nurses were fortified to ameliorate communication in terms of pain management. The pharmacological pre- and postoperative pain management concept was not changed after implementation: Patients received an oral benzodiazepine one hour preoperatively followed by spinal cord anesthesia (4 mL bupivacaine 0.5%, 0.1 mg morphine). Sedation during surgery was maintained with Propofol. Within the first twelve hours postoperatively, patients obtained 3 mg piritramide on demand by an intermediate care nurse. Standard analgesic consisted of Ibuprofen 600 mg (1-1-1) and Metamizole 500 mg (1-1-1-1) daily. Patients may receive additional medication depending on the NRS: Tramadol 100 mg for NRS 3-6, and in case of persistent pain, a repeat dose after 30 min. Oxycodone 20 mg for NRS 7-10 and a repeat dose after one hour. The patients were continuously encouraged to demand additional pain medication and were told not to try to bear the pain. In addition, patients were educated to relieve pain by self-activation and cool packs were provided at any time.

### 2.2. Cessation of the Implementation Study

With cessation of the implementation study in 2016, the interdisciplinary meetings of the health care team and special education for nurses were stopped. However, the operation as well as the pre-and postoperative pharmacological treatment were performed as described above and did not change in comparison to the implementation study. Simple data assessment for the QUIPS project was followed, consisting of baseline data, mean NRS for maximum, minimum and activity-related pain as well as patient satisfaction. A specialized independent pain nurse interviewed the patients 24 h postoperatively and documented postoperative pain management and complications (see below). However, the specialized pain nurse only performed the data assessment and did not take part in pain management. Moreover, the occurrence of possible side effects such as nausea, dizziness, tiredness as well as the use of postoperative nausea and vomiting (PONV) prophylaxis and analgetic consumption was noted. Functional parameters, including pain affected the ability to move, cough, take a deep breath or sleep as well as influencing patients’ mood. Data assessment was performed by aid of a validated 16-item questionnaire, asking for minimal and worst pain since surgery using a numeric rating scale (NRS, 0 = free of pain, 10 = worst imaginable pain). The patients’ questionnaire and the file reporting demographic data are provided under the URL https://www.quips-projekt.de/services/dateien (accessed on 1 April 2023) and attached to the document.

### 2.3. QUIPS Project—Benchmarking and Feedback Concept

Primary data were collected for the QUIPS project, a German-wide benchmarking initiative which compares pain outcome parameters between the 24 contributing hospitals. The other hospitals were anonymous and used their own pain management concept. Being widely accepted and supported by the German Society for Anesthesiologists and the German Society of Surgeons, the project was established and validated by one of our coauthors [5]. All data were anonymized. This study was carried out in accordance with the ethical standards of the Declaration of Helsinki (1975). Patients were informed in written form as well as orally by the study team and informed consent was obtained of all subjects. Participation was voluntary and withdrawal was possible at any time. It was approved by the ethics committee as well as the data security board of Jena University Hospital (Jena, Germany) and the local ethics committee. This study is registered in the DRKS under DRKS00006153 (WHO register).

### 2.4. Statistical Analysis

The Shapiro–Wilk normality test was used to test for normal distribution. Data were not normally distributed. Metric variables are noted as the median ± interquartile range (IQR). Categorical variables are noted in relative frequency. To test for statistical significance, we used the *Chi-square test* and the *non-parametric Mann–Whitney U test*. Statistical significance was considered *p* < 0.05. Significant values were highlighted in italics. Statistical analysis was performed with SPSS (IBM SPSS Statistics 28, International Business Machines Corporation (IBM), Armonk, New York, NY, USA). 

## 3. Results

Altogether, 491 patients at a mean age of 65 years receiving primary THA at our center of excellence for arthroplasty in 2015 and 2021 were included in this study (2015 *n* = 201, 2021 *n* = 290). More than half of the patients met an American Society of Anesthesiologists (ASA) score of two (Table 1). In 2015, significantly more patients had an ASA score of three (*p* = 0.014). THA was performed by senior orthopedic surgeons at a mean duration of 66.5 and 64 min (min). Most patients suffered from chronic pain in the operated region for more than three months preoperatively, meeting a median NRS of seven. In 2021, significantly more patients reported chronic pain, meeting a significantly higher NRS (*p* < 0.001). Demographic data is shown in Table 1.

### 3.1. Postoperative Pain Development

The mean pain (NRS 0–10) was imposed preoperatively as well as minimum, maximum and activity-related pain 24 h postoperatively. The minimum pain did not show significant variation. However, maximum pain and activity-related pain appeared to be significantly higher in 2021 (both *p* < 0.001, see Table 2). The subjective patient participation in pain management was also quantified by an NRS, showing significantly less patient participation in 2021 (*p* < 0.001, see Table 2). The overall satisfaction with the existing pain management concept did not change significantly in comparison to 2015 (*p* = 0.849, see Table 2).

### 3.2. Postoperative PONV Prophylaxis and Analgesics Consumption

In both groups, half of the patients received postoperative nausea and vomiting (PONV) prophylaxis (*p* = 0.927). Non-opioid and opioid consumption in the intermediate care unit (IMC) appeared to be significantly higher in 2021 (*p* = 0.024, *p* < 0.001). Referring to opioid demand, we detected the same difference on ward (*p* = 0.009), while non-opioid consumption was identical. Data of PONV prophylaxis and postoperative analgesics consumption is demonstrated in Table 3.

### 3.3. Occurrence of Side Effects

Nausea was reported by one-fourth of patients and occurred in comparable frequencies in 2015 and 2021. In comparison to 2015, significantly more patients suffered from dizziness (*p* = 0.016), while the frequency of tiredness was reduced (*p* = 0.007). The occurrence of side effects is noted in Table 4.

### 3.4. Functional Outcome after Surgery

The functional outcome after surgery, reflected by the pain affected ability to move, to cough and to sleep as well as the pain affected mood did not show significant differences between the two time points. The functional outcome after surgery is shown in Table 5.

### 3.5. Comparison among 24 Anonymized Hospitals

**Figure 1:**
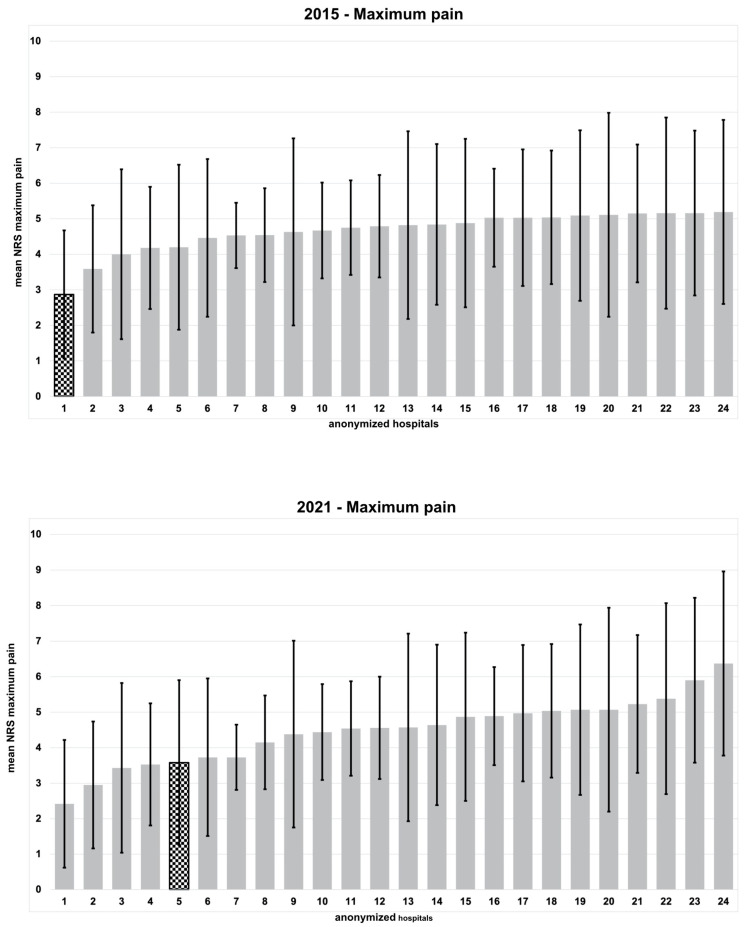
Ranking of maximum pain (mean NRS) 24 h postoperatively among 24 anonymized hospitals from 2015 and 2021. Each bar represents the different mean maximum pain for each contributing hospital, ranging from zero (no pain) to 10 (maximum pain). The checkerboard pattern represents our hospital.

After implementing this special pain management concept in 2014, our clinic showed the lowest maximum pain score in comparison to 49 anonymized hospitals in 2015. In contrast, we dropped behind to fifth place in 2021 in terms of maximum pain. In the meantime, the participation rate in the QUIPS project decreased to only 24 participating hospitals. The comparison with the other anonymized hospitals is illustrated in Figure 1.

## 4. Discussion

Between 2014 and 2015, we implemented a standardized multidisciplinary pain management concept at our tertiary referral center. Featuring a multidisciplinary health care team formed by orthopedic surgeons, anesthesiologists, nurses, and physiotherapists, it aimed to benchmark, detect and erase failures. The nurses received special education in pharmacological and non-pharmacological pain management and their role as the main medical contact person was strengthened. Most deficiencies in pain management appeared to be of organizational issues and a lack of education in pain management which could be solved. Subsequently, we detected constant pain reduction and high patient satisfaction over a period of two years [16]. The implementation study ended in 2016. Thereby, the weekly meetings of the health care team and special education for nurses were stopped, while operation technique, anesthesia and the standard analgesics pain scheme were not changed (see above). In a retrospective study design, we aimed to evaluate if it was possible to obtain the early great results involving low pain scores and low opioid consumption over a period of six years within the daily clinical routine.

The data showed a significant increase in maximum as well as activity-related pain (both *p* < 0.001) in 2021. Farrar et al. defined a change of at least two points on a NRS as clinically relevant, comparing the median [19]. In 2021, opioid consumption was significantly higher in the IMC (*p* < 0.001) as well as on ward (*p* = 0.009). In line with the increment of pain, the subjective patient participation in pain management appeared to be significantly reduced in comparison to 2015 (*p* < 0.001). However, this was not reflected by the overall satisfaction with the pain management concept, which remained high and did not differ significantly in comparison to 2015. Compared to the 24 anonymized hospitals, we fell behind to fifth place in terms of maximum pain while the mean NRS for activity-related pain doubled.

The significantly higher pain scores suggest that awareness of postoperative pain management among hospital staff already declined six years after the implementation study. In our opinion, the missing staff education in terms of pain management as well as ceasing the weekly multidisciplinary meetings of the health care team led to a reduced focus on pain management by the treating staff members. The significantly reduced patient participation in pain management suggests that patients were asked less often if they need additional pain medication or non-pharmacological treatment. In addition, we observed a change in the treating nurses due to SARS-CoV 19 pandemic. Therefore, gained knowledge about pain management could not be passed over. Moreover, some nurses helped out from other departments and were less experienced in the treatment of postsurgical orthopedic patients. *The Lancet* listed primary THA as the most successful operation of the century with excellent long-term results [9], supporting the unaltered high patient satisfaction in 2021. The reduced effort in postoperative pain management resulted in significantly worse postoperative pain scores and was reflected by significantly reduced patient participation in pain management. However, one must admit that the subjective patient participation rate was already very high in 2015, with a mean of 9.97 on NRS. In 2021, patient participation in pain management was still very high, with a mean of 9.83. Therefore, this observation might be statistical relevant but is not from clinical relevance [19]. Another explanation for high patient satisfaction might be the persistent use of spinal cord anesthesia with excellent postoperative pain control. A recent meta-analysis showed significantly better results for spinal cord anesthesia compared to the different pain management interventions for THA as psoas compartment block, periarticular injection, femoral nerve block, lumbar plexus block or local infiltration analgesia [20]. In terms of spinal cord and regional anesthesia, the occurrence of rebound pain as a side effect is often discussed, even though its causes remain mainly unclear [21]. After good initial perioperative pain compensation, the fading away of local anesthesia results in a disproportional excessive increment of pain [21,22]. However, this phenomenon is discussed quite controversially. Recent studies report a prevalence of up to 40%, while in contrast, patients reported high satisfaction [23,24,25]. Regarding pain management, different studies support a multimodal pain management concept such as the one we used [24].

The use of PONV-prophylaxis and occurrence of nausea showed comparable results in 2015 and 2021. The significantly higher appearance of dizziness in 2021 may be a result of the significantly increased opioid consumption. However, the patients reported significantly less tiredness in 2021 (*p* = 0.007). Gentry et al. showed that prior opioid usage is associated with chronic opioid consumption after THA [12]. Although we only evaluated the outcome 24 h after surgery, these findings are in line with our data. We observed significantly higher pain scores and opioid consumption in 2021. Meanwhile, the patients reported chronic preoperative pain significantly more often, in combination with unsignificantly higher opioid usage in comparison to 2015. The different approaches for primary THA are often discussed in terms of postoperative pain outcome. All patients in the present study received an anterolateral approach as described above. A recent study examined if the surgical approach affects chronic opioid usage after THA. The three most common approaches (direct-anterior, antero-lateral and lateral) did not show a significant difference in opioid usage one year after surgery [12].

In recent years, there has been a lot of effort to reduce postsurgical pain [13]. By implementation of enhanced recovery after surgery concepts (ERAS), involving minimal-invasive surgery, early mobilization, preoperative administration of non-steroid-anti-inflammatory-drugs, local-infiltration analgesia, and topical use of tranexamic acid instead of drains, major efforts have been made to improve patient satisfaction and postoperative success [20,26,27]. Moreover, the wider use of spinal and combined spinal and general anesthesia is reported to lead to lower postoperative pain scores in comparison to general anesthesia [28]. Recently, the usage of perioperative dexamethasone administration as part of a multimodal pain management concept is discussed. The authors highlight its positive anti-emetic effect and the possible reduction in opioid consumption and therefore faster rehabilitation [29]. Alternative strategies to reduce chronic pain are widely discussed and examined. A recent meta-analysis states radiofrequency as a promising therapy for chronic musculoskeletal pain by interruption of nociceptive pathways. This tool could especially be used when other technics are not sufficient or not applicable [30]. However, further studies on chronic pain and treatment strategies are needed.

The fact we still maintained an acceptable ranking in comparison to the other clinics while maximum and activity-related pain significantly increased highlights the value of the implemented multidisciplinary pain management concept. It supports the implementation of continuous quality improvement (CQI) strategies to benchmark and ameliorate deficiencies in pain management. However, one has to take into consideration the lower participation rate of hospitals and therefore a possible bias. In 2021, the participation rate in the benchmarking project even decreased, starting with 49 participating clinics in 2015 and more than halved to only 24 clinics in 2021. The collected data with significantly higher pain and less hospital participation in this major benchmarking program highlight the urgent need for high-quality postoperative pain management which can be maintained in addition to the clinical routine. Consisting of three different columns (pre-, intra- and postoperative), adequate pain management for patients receiving THA remains challenging and can only be addressed sufficiently if close patient contact with a special educated health care team is guaranteed. This hypothesis is supported by our data, with significantly worse pain scores already five years after the initial pain management concept was stopped. Furthermore, our data highlight the need for an adequate workforce in the health care system. Referring to the excellent early results we achieved with this standardized multidisciplinary pain management concept in 2015, the worse pain scores and the obvious drop in the participation rate in the benchmarking project should be seen as warning signs and lead to increased effort and awareness in terms of pain management.

The main limitations of the present study are the retrospective study design and therefore limited data availability. As postoperative pain is only imposed 24 h after surgery, long-term pain development and opioid consumption cannot be further evaluated. Therefore, a longer follow-up should be performed in further studies. While the functional outcome was pain on movement, it was not possible to correlate the results with a special score for the hip joint.

## 5. Conclusions

Awareness of postoperative pain management seems to have decreased already six years after implementation. Significantly worse pain scores in combination with higher opioid usage and a lower hospital participation rate highlight the urgent need for high-quality postoperative pain management which can be maintained in addition to the clinical routine. The significantly lower patient participation in pain management is in line with the worse pain scores and indirectly highlights the need for special staff education in pain management. The fact patient satisfaction appeared to remain high and did not differ significantly from 2015, as well as the fact we still achieved an acceptable ranking in comparison to other clinics, highlight the value of the implemented multidisciplinary pain management concept.

## Figures and Tables

**Table 1:** Demographic data.

	2015 (*n* = 201)				2021 (*n* = 290)				
	Median ± IQR		Range		Median ± IQR		Range		*p*-Value
Age (yrs.)	65 ± 20		35–85		65 ± 20		35–85		0.762
Sex (male:female)	95:105				138:151				0.99
Duration of surgery (min)	66.5 ± 23		32–152		64 ± 18		36–145		0.46
ASA Score - Frequency (%)	1	2	3	4	1	2	3	4	
	*16.9*	*54.2*	*28.9*	*0*	*17.6*	*66.6*	*15.9*	*0*	***0.014***
- Absolute number	*34*	*109*	*58*	*0*	*51*	*193*	*46*	*0*	***0.014***
**Pain before surgery**									
Chronic pain >3 months preoperatively % (number)	*92 (185/201)*				*98.6 (286/290)*				***<0.001***
- Operated region (%)	94.1 (174/185)				94.8 (271/286)				0.772
- Operated + 1 other region % (number)	5.9 (11/185)				4.9 (14/286)				0.772
Preoperative opioid consumption % (number)	0 (0/201)				2.1 (6/290)				0.086
NRS chronic pain	*7 ± 2*		*3–10*		*7 ± 3*		*2–9*		***0.017***

**Table 2:** Mean NRS minimum, maximum and activity-related pain, participation in pain management and satisfaction with pain management 24 h postoperatively.

	2015 (*n* = 201)		2021 (*n* = 290)		
	Median ± IQR	Range	Median ± IQR	Range	*p*-Value
NRS minimum	0 ± 0	0–1	0 ± 0	0–1	0.069
NRS maximum	*3 ± 3*	*0–9*	*5 ± 2*	*0–9*	***<0.001***
NRS activity-related	*1 ± 2*	*0–5*	*3 ± 2*	*0–6*	***<0.001***
Participation in pain management	*10 ± 0*	*9–10*	*10 ± 0*	*8–10*	***<0.001***
Satisfaction with pain management	10 ± 0	7–10	10 ± 0	3–10	0.849

**Table 3:** PONV prophylaxis and postoperative analgesics consumption.

	2015 (*n* = 201)	2021 (*n* = 290)	
	Relative Frequencies	Relative Frequencies	*p*-Value
PONV prophylaxis % (number)	53.7 (108/201)	53.1 (153/288)	0.927
IMC non-opioid % (number)	*94 (189/201)*	*97.9 (284/290)*	***0.024***
IMC opioid % (number)	*33.8 (68/201)*	*85.2 (247/290)*	**<*0.001***
ward non-opioid % (number)	100 (201/201)	100 (288/288)	0.99
ward opioid % (number)	*34.8 (70/201)*	*46.9 (135/288)*	***0.009***

**Table 4:** Occurrence of postoperative side effects.

	2015 (*n* = 201)	2021 (*n* = 290)	
	Relative Frequencies	Relative Frequencies	*p*-Value
Nausea % (number)	28.4 (57/201)	26.9 (78/290)	0.756
Dizziness % (number)	*19.9 (40/201)*	*30.0 (87/290)*	***0.016***
Tiredness % (number)	*33.8 (68/201)*	*22.8 (66/290)*	***0.007***

**Table 5:** Functional outcome after surgery.

	2015 (*n* = 201)	2021 (*n* = 290)	
	Relative Frequencies	Relative Frequencies	*p*-Value
Pain affected ability to move % (number)	28.9 (58/201)	25.5 (74/290)	0.469
Pain affected ability to cough/deep breath % (number)	4.5 (9/201)	4.8 (14/290)	0.99
Pain affected ability to sleep % (number)	13.4 (27/201)	9.3 (27/290)	0.186
Pain affected mood % (number)	1.0 (2/201)	1.4 (4/290)	0.99

## Data Availability

On request, data are available from the authors’ institution.
